# Hide and seek: a case of almost missed ingested foreign body

**DOI:** 10.1055/a-2622-4614

**Published:** 2025-07-25

**Authors:** Wenjuan Yang, Yuting Zhao, Jiedong Ma, Jing Li

**Affiliations:** 1 34753 Department of Gastroenterology, West China Hospital, Sichuan University, Chengdu, China

A 33-year-old female was referred to our hospital requiring endoscopic removal of an ingested foreign body. She swallowed a fishbone 1 day ago with a persisting foreign body sensation in the esophagus. Computed tomography reported a high-density shadow about 2 cm in length located at the level of the esophageal entranceway ( Fig. 1 ). Therefore, upper gastrointestinal endoscopy was performed. However, no foreign object or wound was found in the upper gastrointestinal tract after repeated inspection ( Fig. 2 ). Thus, the foreign body was considered to be already discharged into the middle/lower digestive tract, and foreign body removal was given up. Careful observation was still continued until the endoscope was retreated into the mouth. Suddenly, what a surprise, a thin and transparent fishbone was found inserted in the glottis and parallel with the left vocal cord ( Fig. 3 ). Foreign body removal was carried out immediately to prevent its displacement ( Fig. 4 ). The fishbone was successfully removed with biopsy forceps ( Video 1 and Fig. 5 ). Specific foreign body forceps were not used because the foreign body was too thin to be firmly clamped.

**Fig. 1:**
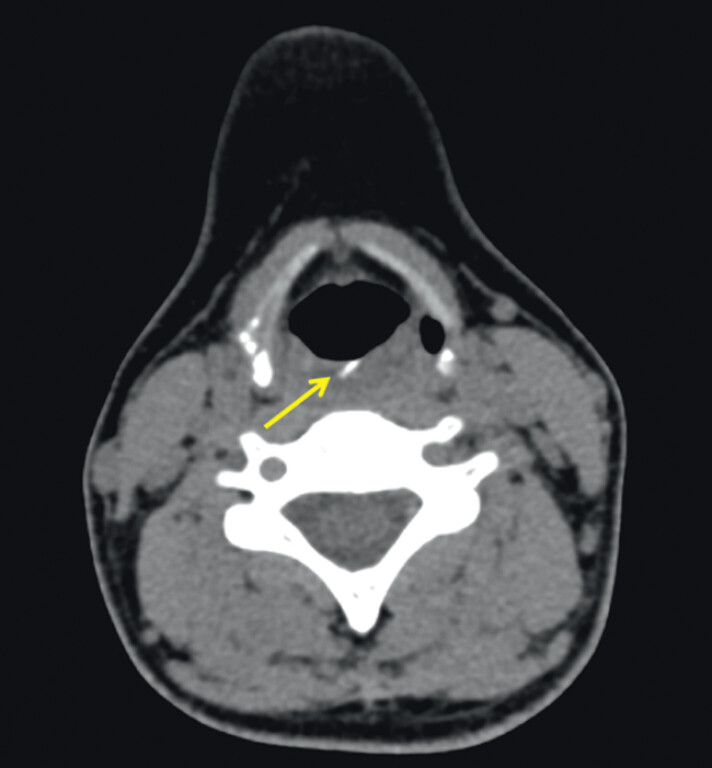
Computed tomography showed a high-density shadow (yellow arrow) about 2 cm in length located at the level of the esophageal entranceway.

**Fig. 2:**
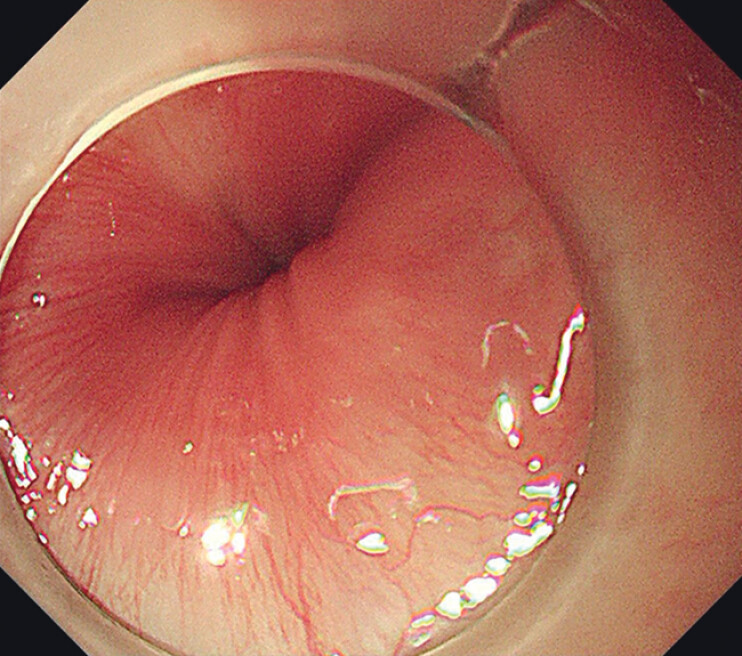
No foreign object or wound was found in the upper gastrointestinal tract under repeated endoscopic inspection.

**Fig. 3:**
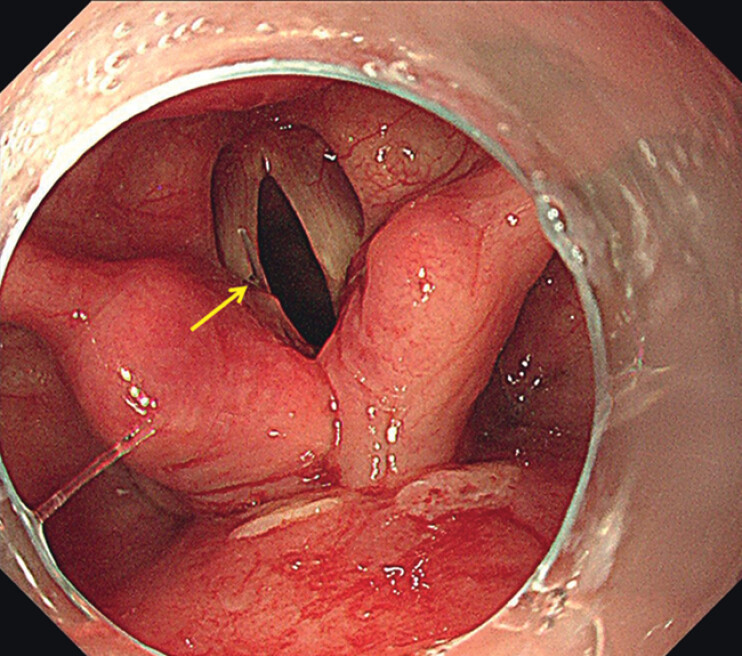
A thin and transparent fishbone about 2 cm in length (yellow arrow) was found inserted in the glottis and parallel to the left vocal cord.

**Fig. 4:**
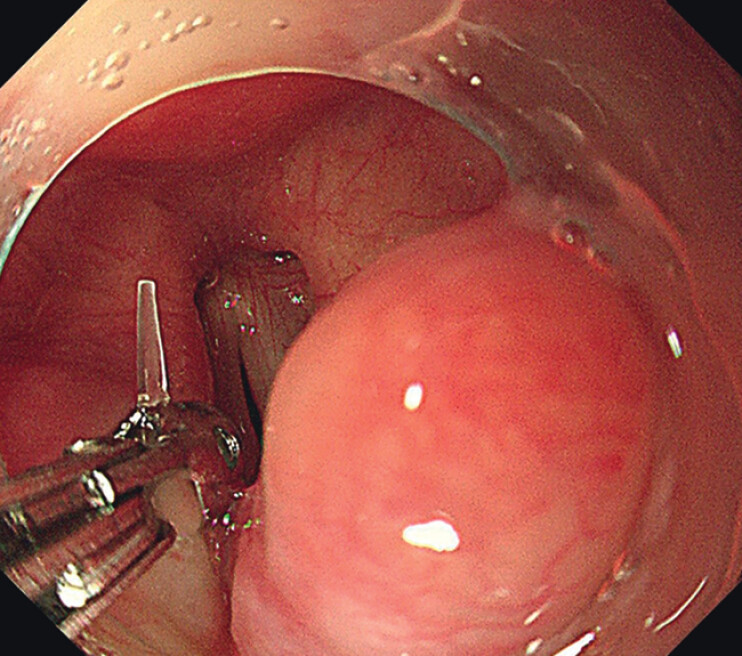
Foreign body removal was carried out immediately with biopsy forceps under digestive endoscopy.

**Fig. 5:**
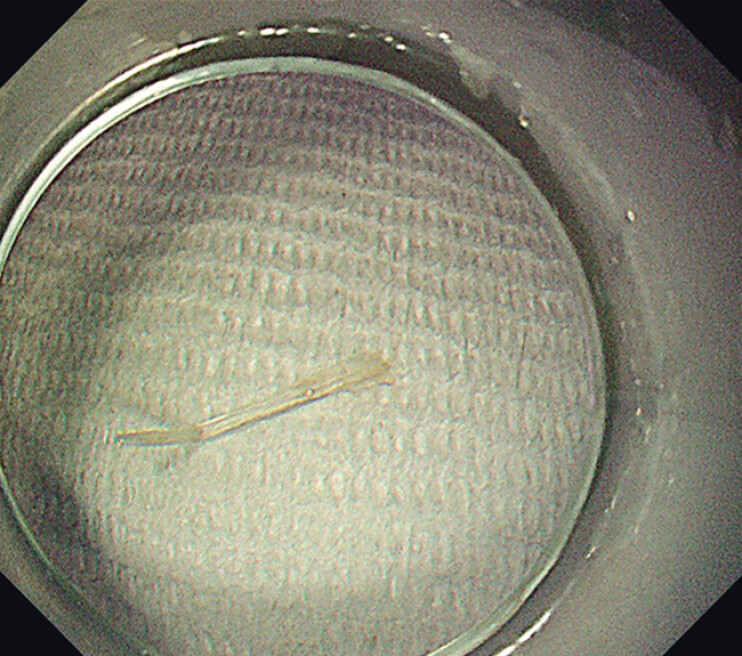
The fishbone at the airway entrance was successfully removed.

Hide and seek: an almost missed airway fishbone foreign body was found and successfully removed with biopsy forceps under digestive endoscopy.Video 1

It is common that the ingested foreign body was not found in the upper gastrointestinal tract during endoscopy when it was discharged into the middle/lower digestive tract or penetrated outside the wall of the digestive tract 1 . In this case, although the foreign body was not found in the upper digestive tract, it was fortunately found at the airway entrance under careful digestive endoscopic observation and successfully removed via a digestive endoscope. This case suggested that the airway entrance and mouth should also be screened carefully during ingested foreign body removal, especially when the foreign body was not found; otherwise, it may cause omission of the foreign body and serious delay in treatment 2 . When a foreign body in a high position of the airway was found during digestive endoscopy, removal may be carried out immediately if a high success rate was assessed.

Endoscopy_UCTN_Code_TTT_1AO_2AL

## Notes

Endoscopy E-Videos https://eref.thieme.de/e-videos
*E-Videos* is an open access online section of the journal *Endoscopy* , reporting on interesting cases and new techniques in gastroenterological endoscopy. All papers include a high-quality video and are published with a Creative Commons CC-BY license. Endoscopy E-Videos qualify for HINARI discounts and waivers and eligibility is automatically checked during the submission process. We grant 100% waivers to articles whose corresponding authors are based in Group A countries and 50% waivers to those who are based in Group B countries as classified by Research4Life (see: https://www.research4life.org/access/eligibility/ ). This section has its own submission website at https://mc.manuscriptcentral.com/e-videos .
